# The Off-Target Cardioprotective Mechanisms of Sodium–Glucose Cotransporter 2 Inhibitors: An Overview

**DOI:** 10.3390/ijms25147711

**Published:** 2024-07-14

**Authors:** Loredana N. Ionică, Adina V. Lința, Alina D. Bătrîn, Iasmina M. Hâncu, Bogdan M. Lolescu, Maria D. Dănilă, Lucian Petrescu, Ioana M. Mozoș, Adrian Sturza, Danina M. Muntean

**Affiliations:** 1 Department of Internal Medicine-Medical Semiotics, “Victor Babeș” University of Medicine and Pharmacy of Timișoara, E. Murgu Sq. No. 2, 300041 Timișoara, Romania; ionica.loredana@umft.ro; 2 Doctoral School Medicine-Pharmacy, “Victor Babeș” University of Medicine and Pharmacy of Timișoara, E. Murgu Sq., No. 2, 300041 Timișoara, Romania; adina.linta@umft.ro (A.V.L.); alina.stanciu@umft.ro (A.D.B.); iasmina-maria.hancu@umft.ro (I.M.H.); bogdan.lolescu@umft.ro (B.M.L.); 3 Centre for Translational Research and Systems Medicine, “Victor Babeș” University of Medicine and Pharmacy of Timișoara, E. Murgu Sq. No. 2, 300041 Timișoara, Romania; danila.maria@umft.ro (M.D.D.); petrescu_lucian@yahoo.com (L.P.); ioanamozos@umft.ro (I.M.M.); daninamuntean@umft.ro (D.M.M.); 4 Department of Functional Sciences-Pathophysiology, “Victor Babeș” University of Medicine and Pharmacy of Timișoara, E. Murgu Sq. No. 2, 300041 Timișoara, Romania

**Keywords:** sodium–glucose cotransporter 2 inhibitors, off-target effects, cardioprotective mechanisms, experimental models, human samples

## Abstract

Sodium–glucose cotransporter 2 inhibitors (SGLT2i), a novel class of glucose-lowering drugs, have revolutionized the management of heart failure with reduced and preserved ejection fraction, regardless of the presence of diabetes, and are currently incorporated in the heart failure guidelines. While these drugs have consistently demonstrated their ability to decrease heart failure hospitalizations in several landmark clinical trials, their cardioprotective effects are far from having been completely elucidated. In the past decade, a growing body of experimental research has sought to address the molecular and cellular mechanisms of SGLT2i in order to provide a better understanding of the off-target acute and chronic cardiac benefits, beyond the on-target renal effect responsible for blood glucose reduction. The present narrative review addresses the direct cardioprotective effects of SGLT2i, delving into the off-target mechanisms of the drugs currently approved for heart failure therapy, and provides insights into future perspectives.

## 1. Introduction

Cardiovascular diseases remain the leading cause of morbidity and mortality worldwide [1] and the ageing of population’s end-stage syndrome, heart failure (HF), is increasingly becoming a challenging issue for healthcare systems [2]. The current classification by the European and American guidelines recognizes three main types of HF according to the left ventricle ejection fraction (EF): HF with reduced EF (HFrEF, EF ≤ 40%), HF with mildly reduced EF (HFmrEF, EF = 41% to 49%), and HF with preserved EF (HFpEF, EF: ≥50%) [3,4]. It has been estimated that HF prevalence will increase from 4.3% in 2010 to 8.5% in 2030 among patients aged 65–70 years, and that the risk for HFpEF will increase in men [5]. The hallmark of its progression is represented by the occurrence of episodes of aggravation, a phase referred to in the literature as worsening HF [6], which renders it the major cause of hospitalizations for the elderly population [7]. Despite the advances made in therapeutics, patients diagnosed with this syndrome still have poor life quality and a poor prognosis [8]. The development of disease-modifying therapies with novel mechanisms of action was an unmet need until the development of sodium–glucose cotransporter 2 inhibitors. Indeed, this class of antidiabetic drugs has increasingly become the cornerstone of HF therapy [9] as a result of the unprecedented positive results of landmark randomized trials, which have demonstrated the reduced hospitalizations for HF and cardiovascular death, regardless of the left ventricular EF status and presence of diabetes (reviewed in [10,11,12]).

Active glucose transport across the epithelial cells of the intestine and kidneys occurs via the sodium–glucose cotransporters (SGLT). The SGLT isoform 1 (SGLT1) is essential for the absorption of glucose in the intestine, while the SGLT isoform 2 (SGLT2) is responsible for glucose reabsorption in the kidney proximal tubules [13]. Almost two decades ago, SGLT2 was discovered and used by the pharmaceutical industry to develop effective antidiabetics drugs, sodium–glucose cotransporter 2 inhibitors (SGLT2i) and gliflozins [14]. This novel class of oral antidiabetics improves the control of blood glucose by inducing glycosuria. These glucose-lowering drugs have revolutionized not only the treatment of diabetes and HF but also chronic kidney disease (CKD). Indeed, canagliflozin, empagliflozin, dapagliflozin and ertugliflozin have been reported by several landmark trials to improve renal outcomes in patients with CKD, with and without diabetes [15,16,17]. Empagliflozin and dapaglifozin have been approved as add-on therapies to the standard HF treatment by the European and American guidelines for HF management [4,18,19]. More recently, sotagliflozin, a dual inhibitor of both SGLT1 and SGLT2, has been approved in the USA to reduce episodes of worsening HF and the risk of cardiovascular death in patients with type 2 diabetes and CKD and in diabetic and non-diabetic patients with HF (regardless of the EF) in the presence of additional risk factors [20]. By inhibiting the SGLT2 isoform in the proximal renal tubule to promote glucose urinary excretion, the SGLT2i reduce glycemia, independent of insulin and with minimal hypoglycemic risk. However, SGLT2i have cardio- and renoprotective effects, in addition to their primary use as pharmacological agents for glucose control in diabetes, effects unrelated to the glycemia improvement [21]. Indeed, several landmark meta-analyses have reported that SGLT2i reduces hospitalizations for HF and other composite endpoints (e.g., cardiovascular death, all-cause death, first and recurrent heart failure hospitalizations, and urgent heart failure visits not requiring hospitalizations), independent of the presence of diabetes and the effects were found to be homogenous across the left ventricle EF strata [22,23]. The absolute benefits were reported to be most pronounced in the first year of treatment, in particular in patients with poorer prognostic (e.g., those newly diagnosed with heart failure in the hospital) [24]. Moreover, a recent meta-analysis has highlighted the time-varying effects of SGLT2 inhibitors, showing that, while the relative reduction in hospitalization for heart failure was significant early on, the benefit tended to decrease over time [25].

In this narrative review, we aim to summarize the mechanistic evidence for the direct cardioprotective effects of SGLT2i in the absence of diabetes, in line with the original and review papers on the topic indexed in the PubMed, Web of Science, and Google Scholar databases. First, we briefly list the systemic effects arising from their “on-target” renal action that lead to cardioprotection and then comprehensively address the “off-target” cardiac effects and the underlying mechanisms relevant for HF. Their cardioprotective effects in animal models of acute myocardial ischemia/reperfusion injury have been well summarized by recent comprehensive reviews [26,27,28]. 

## 2. Systemic Cardiovascular Protective Effects of SGLT2i—A Brief Overview

The systemic hemodynamic and metabolic effects of SGLT2i are directly or indirectly related to glycosuria and natriuresis, which has led to valuable hypotheses of cardiovascular protection that were comprehensively reviewed at the time [29,30,31,32,33,34,35]. The systemic cardioprotection elicited by SGLT2i can be explained (at least partially) according to the following hypotheses and related mechanisms: (i) the “diuretic effect” hypothesis, where osmotic diuresis (due to glycosuria) and increased natriuresis are followed by a subsequent reduction in venous return, preload and ventricular wall stress; (ii) the “blood pressure reduction” hypothesis, where a reduction in circulation volume (via osmotic diuresis), decreased arterial stiffness (due to natriuresis and total sodium reduction) and vasodilation due to both angiotensin II preferentially binding to type AT2 receptors (in the setting of the renin–angiotensin–aldosterone system blockade by the standard HF treatment) and the activation of the angiotensin (1–7) pathway, are both responsible for lowering blood pressure and afterload; (iii) the “anti-inflammatory effect” hypothesis, where decreased plasma levels of uric acid (which activates the NLRP3 inflammasome, increases the levels of pro-inflammatory cytokines and pro-oxidative mediators and lowers the expression of the adhesion molecules) reduces the recruitment of inflammatory cells at the endothelial surface, thus alleviating endothelial dysfunction and molecular changes associated with atherogenesis; (iv) the “metabolic effect” hypothesis, where caloric loss in the urine (200–250 Kcal/day due to glycosuria) leads to weight loss (approx. 2 kg at 6 months and which is persistent) and a negative calorie balance that is associated with a reduction of visceral fat and amelioration of liver steatosis; and (v) the “thrifty substrate” hypothesis, postulating that mice rendered incapable of oxidizing 3-hydroxybutyrate in their hearts exhibited worsened heart failure in response to fasting or a pressure overload, suggesting that the failing heart uses ketone bodies as a metabolic stress defense [36]. Similar to diabetes, where a reduction of the insulin/glucagon ratio will favor increased lipolysis, fatty acid oxidation and ketone production by the liver leads to ketonemia and the preferential utilization of beta-hydroxybutyrate as a ‘super fuel’ by the failing heart [37], in order to provide an extra source of energy [38]. This latter hypothesis has been recently challenged by Avogaro and Del Prato, who have hypothesized that the cardioprotective effects of SGLT2i may be related to their ability to switch cell programming toward a dormancy state (and not a defense state) resembling that of hibernation (where the beta-hydroxybutyrate level is increased and used as key metabolic substrate), in association with the activation of other signal transduction pathways responsible for cellular protection (that are also activated by caloric restriction and exercise) [39].

Nevertheless, given the modest reduction in volemia, blood pressure and body weight due to the on-target effects of SGLT2 inhibitors, additional systemic mechanisms have been postulated to be cumulatively responsible for cardioprotection in the setting of HF, including increased erythropoietin and hematocrit (with hemoconcentration that enhances oxygen delivery to the tissues, including the heart), increased circulating pro-angiogenic progenitor cells and decreased arterial stiffness (thus promoting vascular health), and long-term improvement in renal function due to the beneficial impact on neurohormonal stimulation (e.g., reduction of the sympathetic system activation and subsequently of pulmonary pressure). 

## 3. Cardiac Protective Effects of SGLT2i

SGLT2i elicit cardioprotective effects, which are independent of their interaction with the SGLT2 receptors and anti-diabetic action, termed “off-target” effects, that have been unequivocally demonstrated in several preclinical animal models (reviewed in [40]), including in animals lacking the renal SGLT2 expression [41]. The mechanisms behind their “off-target” beneficial cardiac effects remain a partially elucidated ‘mystery’ [42,43], mainly because most previous studies, with few recent exceptions [44,45], were able to detect the SGLT1, but not the SGLT2 expression, in either normal or diseased human hearts [46,47,48,49,50,51].

We summarize below the direct mechanisms responsible for the cardioprotection elicited by the most investigated SGLT2i drugs, in the absence of diabetes, in particular for those currently approved for the treatment of HF; their effects in the diabetic hearts are well covered in [52,53,54]. As such, the “off-target” cardiac effects of empagliflozin (EMPA), dapagliflozin (DAPA), and canagliflozin (CANA) are systematically categorized according to their impact at the organ level, as well as at cellular and molecular levels. Additionally, the current data regarding the protection afforded by the dual SGLT2/1 inhibitor sotagliflozin (SOTA) are presented separately. Ultimately, we address the emerging role of SGLT1 inhibition in cardioprotection.

## 4. Cellular and Molecular Effects

### 4.1. Protection of Mitochondrial Function and Structure

Mitochondrial dysfunction has been systematically implicated in the development of cardiac diseases [55], including heart failure [56]. In the past decade, an impressive number of experimental studies have addressed the role of SGLT2i in improving mitochondrial bioenergetics, dynamics, biogenesis, and mitophagy in various organs, cell lines and experimental setups, mainly in the presence of diabetes [57]. Notably, a recently postulated mechanism by which SGLT2i may retard the progression of HF by modulating the mitochondrial function is the augmentation of cytosolic Fe^2+^, which is likely to increase the ATP production of the cardiomyocytes [58]. We present below the studies that demonstrate the cardioprotective effects of SGLT2i at the mitochondrial level in the absence of diabetes or other metabolic pathologies.

#### 4.1.1. Empagliflozin

Li et al. evaluated the impact of EMPA on mitochondrial function and oxidative stress in the setting of a non-diabetic mouse model of HF induced by transverse aortic constriction (TAC). They administered EMPA for 4 weeks, starting 2 weeks post-TAC and reported that the drug improved mitochondrial oxidative phosphorylation (OXPHOS), enhanced mitochondrial biogenesis, and restored normal mitochondria morphology. Moreover, EMPA treatment effectively suppressed cardiac ROS production, boosted the expression of endogenous antioxidants, promoted autophagy, and reduced cardiac apoptosis in TAC-induced HF. Additionally, while EMPA treatment did not affect blood glucose or body weight, it did significantly mitigate TAC-induced cardiac dysfunction and ventricular remodeling. Importantly, ex vivo EMPA treatment has been found to increase mitochondrial respiration in cardiac fibers, pointing to a direct cardiac beneficial effect of the drug in non-diabetic pressure overload-induced HF [59].

Shiraki et al. explored the mechanisms by which chronic administration of EMPA affects HF progression in a murine model deficient for heart and skeletal muscle-specific manganese superoxide dismutase that mimic dilated cardiomyopathy. The EMPA group exhibited upregulation of energy metabolism, reduction of cardiac fibrosis and improved survival rate. Specifically, the capacity for oxidative phosphorylation of cardiac mitochondria in the EMPA group was significantly increased and blood lactate was significantly lower, indicating reduced anaerobic glycolysis [60].

Cai et al. used the in vivo model of murine myocardial ischemia/reperfusion (I/R) injury to evaluate the effects of chronic oral EMPA administration (7 days prior to surgery) on isolated cardiac microvascular endothelial cells (CMECs). While the I/R protocol activated mitochondrial fission, oxidative stress and apoptotic signaling in CMECs, EMPA attenuated the I/R-induced cardiac microvascular damage, i.e., normalized mitochondrial fission and fusion, neutralized the supraphysiologic ROS production and suppressed mitochondrial apoptosis. These authors described the signal transduction of these protective effects, which was the activation of mitophagy via the adenosine monophosphate-activated protein kinase (AMPK)α1/Unc-51-like autophagy activating kinase 1 (ULK1) pathway [61].

Lyu et al. assessed the effect of chronic EMPA administration in a murine model of HF induced via left anterior descending coronary artery ligation. Mitochondrial function was assessed by observing mitochondrial morphology, measuring mitochondrial dynamics-related proteins (e.g., mitochondrial fission inhibitor, mdivi1) and analyzing the ATP levels. EMPA inhibited mitochondrial fission and improved energy metabolic efficiency (ATP production) in HF mice by regulating the expression of mitochondrial dynamics-related proteins. By modulating mitochondrial dynamics in HF mice, EMPA also alleviated myocardial fibrosis and cardiac dysfunction [62].

#### 4.1.2. Dapagliflozin

Methamphetamine (METH) causes cardiovascular toxicity, partly by damaging mitochondrial function. He et al. aimed to evaluate DAPA’s ability to prevent METH-induced experimental cardiomyopathy. Mice treated with METH showed mitochondrial dysfunction leading to apoptosis, decreased cardiac function, and increased fibrosis. DAPA significantly improved mitochondrial dynamics and reduced total ROS level and apoptosis, thus improving cardiac dysfunction and fibrotic remodeling [63].

#### 4.1.3. Canagliflozin

Harris et al. used high-sensitivity proteomic techniques to characterize the specific molecular pathways affected by chronic administration (5 weeks) of CANA in a swine model of chronic myocardial ischemia induced by left circumflex coronary artery constriction. Three hundred five proteins were found to be statistically different between the groups, 55 proteins being downregulated and 250 upregulated by the CANA treatment. Pathway analysis revealed the elevation of multiple proteins associated with metabolism and redox activity in the treated group. Notably, at the mitochondrial level, CANA induced a significant increase in the mitochondrial complexes I, II, III, and IV. Furthermore, functional assessment demonstrated a notable rise in cardiac index within the CANA-treated group as compared with controls [64].

### 4.2. Alleviation of Oxidative Stress and Inflammation

Oxidative stress and low-grade inflammation reciprocally reinforce themselves in the pathophysiology of most chronic diseases associated with ageing (hence, the term ‘inflammageing’) and are also bi-directionally related to mitochondrial dysfunction. Thus, all three pathomechanisms contribute to the progression of HF in a dynamic vicious circle [65].

In HF, chronic oxidative stress is linked both to an elevation in ROS levels due to the dysfunctional electron transport system (ETS) and to an increase in the activity of several enzymes such as NADPH oxidases 2 and 4 (Nox2 and Nox4, the latter controversially), monoamine oxidase isoform A (MAO-A), uncoupled mitochondrial NO synthase (mtNOS) and a reduction of the antioxidant defense systems (e.g., mitochondrial superoxide dismutase/mt SOD). The increased release of ROS from the mitochondria into the cytosol may trigger further ROS generation by several mechanisms, including cytosolic NOS uncoupling or conversion of xanthine dehydrogenase to the ROS-producing form of xanthine oxidase. Oxidative stress is further responsible for structural cardiac damage leading to (i) amplification of local and systemic inflammatory state; (ii) stimulation of hypertrophy, fibrosis, and pathological remodeling; (iii) induction of apoptosis; and (iv) impaired calcium regulation, diastolic and systolic dysfunction and increased arrhythmogenesis [66].

SGLT2i also provides indirect cardioprotection by beneficial changes at the level of the epicardial adipose tissue, the fat depot with unrestricted contiguity with the heart and high pro-inflammatory and proarrhythmic profile in the setting of HF. The SGLT2i-related protection has ascribed to the suppression of epicardial inflammation, oxidative stress and adipogenesis [67,68] and has been recently reviewed [69]. Additionally, the role of gliflozins as anti-inflammatory molecules and the experimental evidence in various cell lines and animal models of diabetes has been recently addressed by recent comprehensive reviews [70,71,72].

We further present relevant information related to their cardiac anti-oxidative and anti-inflammatory effects as evidenced in cell lines, animal models of HF or cardiac human samples from failing hearts.

#### 4.2.1. Empagliflozin

Jason Dyck et al. demonstrated that EMPA attenuates the activation of the NLRP3 inflammasome in non-diabetic mice with either HFrEF or HFpEF in a Ca^2+^-dependent manner, as its effect was completely blunted by a calcium ionophore [73].

Koyani et al. evaluated the anti-inflammatory pathways affected by EMPA in vitro using lipopolysaccharide (LPS)-treated HL-1 cardiomyocytes (of mouse atrial origin) and assessed, in vivo, its cardioprotective effects in a mouse model of endotoxaemia. In cardiomyocytes, EMPA attenuated LPS-induced TNFα and iNOS expression and activated AMPK, which subsequently prevented the ATP depletion. In vivo administration of LPS impaired cardiac contractility, while co-administration of EMPA preserved cardiac function, together with improving cardiac AMPK phosphorylation and ATP/ADP ratio and reducing cardiac iNOS, TNFα and creatine kinase MB plasma levels [74]. 

Quagliariello et al. investigated the mechanism by which EMPA exerts anti-inflammatory and cardioprotective effects in a model of doxorubicin-induced cardiotoxicity. First, they used the HL-1 cell line (of mouse cardiomyocytes) for preliminary cellular research and reported that EMPA decreased intracellular ROS and lipid peroxidation. In vivo treatment reduced ferroptosis, fibrosis, apoptosis, and inflammation in the non-diabetic C57BL/6J mice treated with doxorubicin by engaging NLRP3- and MyD88-related pathways, with significant improvements in cardiac function [75]. 

Recently, Li et al. assessed the direct effects of EMPA on high glucose-induced oxidative stress in primary cardiomyocytes from neonatal rats and the related signal transduction. In cells exposed to hyperglycemia (30 mmol/L), EMPA significantly attenuated oxidative stress, cardiomyocyte apoptosis and mitochondrial injury. The effects were mediated by increased protein levels of phosphorylated AMPK and peroxisome proliferator-activated receptor-γ coactivator-1α (PGC-1α) and were partially reversed by the addition of Compound C (the AMPK inhibitor). Importantly, the effects were independent of SGLT2 inhibition as no SGLT2 expression was detected on cardiomyocytes [76]. 

As regards the effects on human samples, Kolijn et al. investigated the anti-oxidant and anti-inflammatory effects of EMPA on myocardial samples, skinned fibers prepared from left ventricle (LV) biopsies that had been harvested from patients with HFpEF. Acute treatment (60 min. incubation) with EMPA significantly decreased the levels of H_2_O_2_, 3-nitrotyrosine, glutathione (GSH), and lipid peroxidation in both cytosol and mitochondria. Moreover, the HF-related increase in oxidative stress diminished NO levels, sGC activity, cGMP concentration, and PKGIα activity (eNOS lead to PKGIα oxidation). EMPA reduced PKGIα oxidation and increased its activity with the improvement of the myofilament protein phosphorylation. The reversal of the NO–sGC–cGMP–PKG cascade resulted in the alleviation of cardiomyocyte stiffness (which can be translated in the improvement in diastolic dysfunction in the clinical arena). Moreover, incubation with EMPA reduced the markers of microvascular inflammation, namely ICAM-1, VCAM-1, TNF-a, and IL-6, whose levels were increased in the setting of HFpEF [77].

#### 4.2.2. Dapagliflozin

Tsai et al. have reported that DAPA attenuates oxidative stress in cardiac myoblast H9c2 cells and primary neonatal cardiomyocytes via Nox2 inhibition mediated by the AMPK/PKC pathway. Treatment with DAPA of H9c2 cells subjected to a 1 h hypoxia and 4 h reoxygenation (H/R) protocol resulted in a time-dependent increase in the expression of phosphorylated AMPK associated with reduction in protein kinase C (PKC) phosphorylation. These authors further dissected the signaling pathway and showed that exposure to the H/R protocol increased the expression of Nox2 and its additional protein subunit Rac-1 in H9c2 cells in a PKC-dependent manner. Accordingly, DAPA protected both H9c2 cells and cardiomyocytes against H/R injury-related oxidative stress through the modulation of AMPK/PKC/Nox2 signaling. In addition, DAPA prevented the H/R-induced abnormal PGC-1α expression, mitochondrial membrane potential, and mitochondrial DNA copy number through AMPK/PKC/Nox2 signaling, which also reversed the H/R-induced apoptosis [78].

Hsieh et al. have demonstrated that DAPA can mitigate Doxorubicin cardiotoxicity by reducing oxidative stress, inflammation, mitochondrial dysfunction, hypertrophy and fibrosis via PI3K/AKT/Nrf2 signaling. In cardiac H9c2 myoblasts treated with Doxorubicin, DAPA induced the activation of AKT/PI3K signaling, leading to the upregulation of the antioxidants hemoxygenase-1 (HO-1), NAD(P)H dehydrogenase quinone 1 (NQO1), and superoxide dismutase (SOD), as well as to an improved mitochondrial dysfunction via the nuclear factor erythroid-2 related factor 2 (Nrf2). In addition, the reduced oxidative stress resulted in the downregulation of the markers of hypertrophy (natriuretic peptides, ANP and BNP) and fibrosis (phospho-Smad3, collagen I, fibronectin, and α-SMA). Furthermore, DAPA also inhibited the inflammatory IL-8 via the PI3K/AKT/Nrf2/p38/NF-κB signaling [79].

In a recent investigation, Wu et al. demonstrated that DAPA exhibits efficacy in reducing HFrEF stemming from both myocardial infarction (MI) and transverse aortic constriction (TAC) and showed that SGLT2 global knockout mice could not be protected from heart failure. DAPA directly inhibited macrophage inflammation and suppressed cardiac fibroblasts activation, thus preventing matrix remodeling. The beneficial effects were blunted in mice treated with a C–C chemokine receptor type 2 antagonist. Consequently, the authors inferred that DAPA’s anti-fibrotic and cardioprotective properties stem from its ability to inhibit the pro-inflammatory progression of macrophages and are independent of SGLT2 [80]. 

We have recently demonstrated that acute ex vivo incubation of right atrial appendages isolated from overweight, nondiabetic patients with all-spectrum HF undergoing cardiac surgery, with either EMPA or DAPA at clinical relevant concentrations, decreased the expression of the mitochondrial enzyme monoamine oxidase (MAO) and alleviated oxidative stress elicited by the ex vivo exposure to angiotensin II (Ang II) and high glucose, as a novel “off-target” cardiac class effect of SGLT2i. Castoldi et al. have reported that 2 weeks administration of EMPA prevented the onset of myocardial hypertrophy and fibrosis in rats with Ang II-induced hypertension by mitigating the cardiac inflammatory response and reducing the expression of tyrosine hydroxylase, a marker of local sympathetic activity [81], and the enzyme catalyzing the first step in the synthesis of catecholamines, the MAO substrates. Similarly, 2 weeks administration of DAPA significantly reduced elevations of tyrosine hydroxylase and norepinephrine levels in kidneys of neurogenic hypertensive mice [82].

#### 4.2.3. Canagliflozin

Hasan et al. showed that isoprenaline administration causes pro-oxidative changes in the heart by stimulating the production of reactive oxygen species and nitrogen species. Chronic CANA administration in isoprenaline-treated non-diabetic rats not only preserved the endogenous antioxidants, but also reduced the markers of cardiac oxidative stress, fibrosis, and apoptosis. Western blot analysis and quantification of mRNA expression have demonstrated that CANA enhances the antioxidant and anti-inflammatory signaling involving AMPK, eNOS, Nrf2, and HO-1. In addition, CANA treatment attenuates the pro-oxidative, pro-inflammatory, and pro-apoptotic signaling mediated by inducible nitric oxide synthase (iNOS), transforming growth factor beta (TGF-β), and Nox4 [83].

More recently, Harris et al. explored the impact of CANA therapy on metabolic pathways and inflammation in the swine model of chronic myocardial ischemia. These authors report the suppression of fatty acid (FA) oxidation and improvement of insulin signaling in ischemic myocardium. Intriguingly, CANA elevates the levels of certain inflammatory cytokines IL-6, IL-17, IFN-gamma and of iNOS associated with a trend toward the decrease of the anti-inflammatory markers IL-10 and IL-4, an observation that needs to be confirmed in humans [84].

## 5. Whole-Organ Effects

### 5.1. Improvement of Cardiac Metabolism

Targeting myocardial substrate metabolism as a therapeutic approach in HF includes the inhibition of myocardial fatty acid uptake and oxidation and an increase in glucose and ketone oxidation. The therapeutic potential of ketones in HF therapy has been well reviewed [85,86]. Ketone therapy is currently investigated as a therapeutic approach in HF not only because it provides an alternative fuel for the energy-starved ‘engine’ but also due to other pleiotropic effects of *β*-hydroxybutyrate (βOHB) [87], as further described.

#### 5.1.1. Empagliflozin

Yurista et al. reported, in non-diabetic rats with post-infarct LV dysfunction, that either early (prior to surgery) or late (2 weeks after surgery) administration of EMPA improved cardiac metabolism and ATP production. EMPA increased circulating ketone levels and myocardial protein expression of the ketone body transporter and of succinyl-CoA:3-ketoacid CoA transferase and *β*-hydroxybutyrate dehydrogenase, two critical ketogenic enzymes. The authors concluded that ATP restoration by EMPA was, at least partially, caused by the increased myocardial utilization of ketone bodies [88].

Chase et al. have hypothesized that EMPA directly interferes with cardiac ketone metabolism independent of the substrate supply, and demonstrated, in a Langendorff-perfused rat heart subjected to ischemia/reperfusion injury, a switch in metabolism away from glucose towards increased ketone oxidation together with an increase in total ATP and phosphocreatine levels [89].

Santos-Gallego et al. further confirmed, in an elegant model of non-diabetic porcine HF, that the cardiac advantages of EMPA stem from its ability to redirect myocardial fuel metabolism away from glucose towards ketone bodies. These authors reported, in a large animal model, that EMPA-treated pigs show a reduction in myocardial glucose uptake and glycolysis-related enzymes, together with a switch toward the utilization of ketone bodies, free fatty acids and branched-chain amino acids. They also showed an increase in both substrates’ uptake and activity/expression of the enzymes belonging to the metabolic pathways, ultimately augmenting myocardial energy production and cardiac function [90].

Jason Dyck et al. also assessed the effect of chronic (2 weeks) treatment with an exogenous ketone ester in mice subjected to TAC and reported a significant alleviation of cardiac dysfunction, hypertrophy and fibrosis as compared with the group treated with the vehicle [91]. 

However, despite these promising experimental data, the recent results of the EMPA-VISION trial, which randomized patients with HFrEF and HFpEF to EMPA (10 mg/day for 12 weeks) vs. placebo and assessed the changes in both cardiac phosphocreatine:ATP ratio at rest and during peak dobutamine stress (using phosphorus magnetic resonance spectroscopy) and serum metabolomics (by mass-spectrometry), were disappointing. Three months treatment with EMPA neither improved cardiac energetics nor changed circulating serum metabolites associated with energy metabolism in patients with HFrEF and HFpEF vs. placebo [92].

Nevertheless, the advantages brought about by increasing ketone plasma levels may not only stem from enhanced energetics, as ketones have been reported to exert other adaptive effects aimed at defending the body against metabolic stress.

As such, besides the beneficial effect on cardiac energy production, the ketone body βOHB, whose level is increased by SGLT2i in both healthy and diabetic mice [93], has been reported to suppress sympathetic activity and decrease norepinephrine release from the adrenergic nerve endings [94]. A lower level of catecholamines after EMPA treatment was also found in a large animal model of HF [90]. Kimura et al. reported in 2011 that βOHB directly suppresses sympathetic activity via the G protein-coupled receptor 41 (GPR41) at the level of the sympathetic ganglia, while, in contrast, propionate (a short-chain fatty acid) activated GPR41, increasing the sympathetic outflow [95]. Moreover, βOHB was reported to exert an anti-inflammatory effect by suppressing the NLRP3 inflammasome activation and the related IL-1β and IL-18 production in human monocytes [96].

In a mouse model of HFpEF, Deng et al. confirmed the fact that βOHB mitigates the NLPR3 inflammasome and alleviates the mitochondrial hyperacetylation elicited by the disease. The authors postulate that the ketone decreased acetyl-CoA via the activation of citrate synthase and suppression of fatty acid uptake, with a subsequent reduction in the mitochondrial protein acetylation [97].

Finally, βOHB is a signaling metabolite that acts as an endogenous inhibitor of histone deacetylases (HDAC) [98]. Shimazu et al. have demonstrated that inhibition of class I HDAC increases histone acetylation at the Foxo3a and *Mt2* promoters, with subsequent protection against oxidative stress, in kidneys isolated from rats treated with paraquat, by suppressing both protein carbonylation and lipid peroxidation [99].

#### 5.1.2. Dapagliflozin

In the DEFINE-HF trial, Selvaraj et al. performed targeted metabolomics in patients with HFrEF treated with DAPA and showed increased plasma levels of ketone bodies (most cases at physiological levels) and free FA-related metabolites. These findings are consistent with the increase in myocardial utilization of ketone bodies by the SGLTi (assessed as increased medium-chain acylcarnitines) as such a cardiac uptake of β-hydroxybutyrate is directly proportional to its blood levels [100].

Notably, in past years the hypothesis of preferential ketone oxidation by the heart (instead of glucose) has become controversial (reviewed in [101]). Most recently, Gary Lopaschuk et al. investigated, in mice that underwent TAC to induce HFrEF, the effects of chronic DAPA administration on cardiac function (4 weeks to increase ketone in serum) and also, in acute settings, in isolated working hearts perfused with glucose, palmitate, and a low or high concentration of βOHB, on energy metabolism. In the chronic model, they reported that glucose oxidation was significantly decreased in TAC vehicle hearts as compared with sham hearts, yet did not further decrease in TAC DAPA hearts, despite an increase in βOHB oxidation in both groups. In the acute model, increasing βOHB supply selectively lowered the fatty acid (but not glucose) oxidation rates. The authors concluded that increasing ketone concentration significantly increases ATP production in HFrEF without further impairing glucose oxidation [102]. In line with this, Douglas Lewandowski et al. reported, in the same experimental model of TAC-induced hypertrophy in rat, that short-chain fatty oxidation outpaced ketone oxidation (at the expense of long-chain fatty oxidation) [103].

The most recent hypothesis with regard to how SGLT2i modulate energy metabolism has been formulated by Milton Packer, who supports the concept that SGLT2 acts as a nutrient surplus sensor; its inhibition causing simultaneous upregulation of the nutrient deprivation signaling (and downregulation of the surplus signaling) manifested by increased activation of AMPK, sirtuin 1, 3 and 6 (SIRT 1, 3, 6) and peroxisome proliferator-activated receptor γ coactivator 1-α (PGC1-α) and by decreased activation of the mammalian target of rapamycin (mTOR). Activation of SIRT1 and AMPK signaling stimulates autophagy and also occurs during the adaptive response to starvation/cellular stress. Besides the activation of the SIRT1/AMPK pathway, SGLT2i also suppress Akt/mTOR signaling, inducing a state of fasting mimicry, which is considered to be responsible for most of the beneficial effects reported for these drugs, e.g., the reduction in oxidative stress and inflammation, normalization of mitochondrial structure and function, contractile performance enhancement etc. Importantly, the SGLT2i-related autophagy augmentation is seen in isolated cells and tissues that do not express SGLT2 and has been ascribed to their ability to bind directly to sirtuins or mTOR. In accordance with this, the cardioprotective effects of SGLT2i are abolished by the specific inhibition/knockdown of autophagy, AMPK, and sirtuins [104].

Moreover, SIRT-1 activation by SGLT2i in turn activates the hypoxia-inducible factor-2α (HIF-2α), which is a major stimulus for the synthesis of erythropoietin with erythrocytosis as an organ-protective mechanism. Notably, in large-scale trials, erythrocytosis has been shown to be the most powerful predictor of SGLT2i beneficial effect of reducing HF events [105].

### 5.2. Cardiac Function Enhancement and Arrhythmia Prevention

#### 5.2.1. Empagliflozin

The first observation that EMPA can positively influence cardiac function in the non-diabetic mice subjected to chronic and acute conditions of pressure overload (despite no clear evidence of molecular targets in cardiac tissue), comes again from Jason Dyck et al. These authors have demonstrated that in vivo administration of EMPA for 2 weeks in the TAC model of HFrEF, and ex vivo treatment of isolated working rat hearts results in an improvement in cardiac systolic (but not diastolic) function. Additionally, no significant changes in myocardial remodeling were observed after this short period of treatment [106].

Lee et al. used a rat model of hypertension-induced HF to confirm that chronic administration of EMPA (12 weeks) ameliorated left atrial dilation, systolic dysfunction and intracardiac fibrosis and also exerted beneficial effects on systemic blood pressure and renal function [107].

Connelly et al. demonstrated that EMPA administration (4 weeks) in a rat model of HFpEF reduced left ventricular mass and improved both wall stress and diastolic function with no difference in the plasma level of beta-hydroxybutyrate vs. the control group [108]. The same group further hypothesized that chronic administration (6 weeks) of EMPA may improve intrinsic cardiac function, independent of the loading conditions in a rat model of HFrEF. They have reported that the load-independent parameters of cardiac contractility, namely, preload, recruitable stroke work, and the end-systolic pressure volume relationship, as well as systolic blood pressure, were higher in EMPA-treated rats vs. control. A trend toward diastolic function improvement (reduced LV end-diastolic pressure) was also recorded [109].

Li et al. also investigated the effect of chronic EMPA treatment (4 weeks) in mice with TAC-induced heart failure and reported the alleviation of both LV systolic and diastolic dysfunction and an increased exercise endurance and survival rate. Additionally, a significant attenuation of adverse ventricular remodeling and cardiac fibrosis was found [110].

Goerg et al. explored the short-term effects of treating normoglycemic rats with EMPA in low dose (1 mg/kg). One week of oral therapy with a low-dose EMPA enhanced fractional shortening, stroke volume, and cardiac output. In a similar manner, after acute administration of EMPA (30 min, intravenous) in healthy animals, the ventricular systolic pressure, mean pressure, and maximum dP/dt were elevated. Additionally, in H9c2 cardiac cells, EMPA prevented apoptosis and decreased the activity of matrix metalloproteinases MMP 2 and 9 [111].

Pabel et al. were among the first to address the direct myocardial effect of EMPA in ventricular samples isolated from patients with end-stage HFpEF. In human failing cardiomyocytes, EMPA influenced neither the calcium transient amplitude nor the level of diastolic calcium; however, in myocardial trabeculae, EMPA reduced myofilament passive stiffness via the enhancement of phosphorylation levels of myofilament regulatory proteins. In rats with HFpEF, intravenous injection of EMPA significantly improved diastolic function while systolic contractility was unaffected [112].

More recently, Axelsen et al. have reported the beneficial effect of EMPA in an animal model of right ventricular (RV) failure induced by pressure overload. These authors investigated the effects of the chronic administration of EMPA (5 weeks) after RV dysfunction induced by pulmonary trunk banding in male rats. A decreased RV end-systolic pressure, without any changes in RV cardiac output or ventricular-arterial coupling, associated with a slight reduction in RV cross-sectional area was found. The authors concluded that further investigations are required to differentiate between the diuretic effect of the drug and its independent beneficial effect on RV function [113].

In the evolution of HF, the impairment of ion homeostasis can lead to electrical disturbances and increase the risk of cardiac arrhythmias. The anti-arrhythmic effect of the SGLT2i may involve several cellular pathways regulating myocardial ion channels and transporters. Their modulation by the SGLT2i has been recently reviewed [114]. Additionally, the evidence for a reduced risk for atrial fibrillation and flutter from clinical trials with SGLT2i have been well covered in a recent review [115] and in two metanalyses [116,117].

In particular, inhibition by EMPA of the sodium–hydrogen exchanger isoform 1 (NHE1, whose activity increases in the setting of HF) and the underlying signal transduction, have been systematically dissected under various experimental conditions and in different species and reported to be cardioprotective by Coert Zuurbier et al. and others (recently summarized in [114,118]), but not by Michael Shattock et al. [119]. The reduced risk of arrhythmia can be ascribed to the mitigation of intracellular sodium and calcium levels, the consequent alleviation of myocardial hypertrophy, fibrosis, and adverse remodeling. However, NHE1 inhibition, the subsequent reduction in cardiac sodium and calcium and the antiarrhythmic effect might be also the consequences of the inhibition by EMPA of Ca^2+^/calmodulin-dependent kinase II (CaMKII), which is upregulated in HF [101].

#### 5.2.2. Dapagliflozin

In the TAC mouse model of HFrEF, DAPA has been shown to be successful as a cardioprotective molecule by improving cardiac systolic function and inhibiting myocardial fibrosis and myocyte apoptosis [120].

Cappetta et al. tested the effects of chronic treatment with DAPA (6 weeks) in a non-diabetic model of HFpEF, the Dahl salt-sensitive rats were fed a high-salt diet to induce hypertension and diastolic dysfunction. They showed that the drug improved diastolic function, while blood pressure remained markedly elevated. As the drug reversed the deficit of the endothelial NO synthase, with subsequent attenuation of cardiac inflammation and pro-fibrotic signaling. The authors concluded that the alleviation of the endothelial coronary artery dysfunction, besides the previously mentioned benefits, translated into improved myocardial performance [121].

In a rat model of mitral regurgitation-induced HF, DAPA reduced left ventricular dysfunction, restored the end-systolic pressure–volume relationship, and decreased the inducibility and duration of atrial fibrillation. Its pleiotropic effects involved the modulation of several pathophysiological mechanisms, such as cardiac fibrosis, cardiac endoplasmic reticulum stress and apoptosis, electrical conduction, and calcium signaling [122].

Lahnwong et al. assessed the effects of DAPA administration before an ischemia/reperfusion protocol in rats, and found better LV function, fewer arrhythmias, reduced necrosis and apoptosis, and improved function, biogenesis, and dynamics of cardiac mitochondria [123].

Thus, in a very recent study, Paasche et al. performed patch clamp experiments on isolated atrial cardiomyocyte and human-induced pluripotent stem cell-derived cardiomyocytes in order to investigate the direct electrophysiological effects of DAPA. In both cases they found a significant reduction of the action potential inducibility and the amplitude plus maximum upstroke velocity, mainly in atrial cells. The latter demonstrated that DAPA directly interferes with the depolarization phase by significantly decreasing the peak sodium current densities accompanied by a moderate inhibition of the transient outward potassium current in human atrial cardiomyocytes. This leads to cardioversion of acute atrial fibrillation episodes to sinus rhythm and rhythm control of persistent atrial fibrillation in a large-animal model (pigs) in vivo. Their results show that DAPA elicits an acute class I antiarrhythmic effect, suggesting a possible role for SGLT2 inhibitors as antiarrhythmic medicines [124].

#### 5.2.3. Canagliflozin

Research has demonstrated that CANA treatment preserves cardiac contractile function and efficiency during acute regional myocardial ischemia in normal, metabolically healthy pigs through acute effects on cardiac volume regulation that cannot be explained by changes in myocardial substrate [125].

In a recent investigation, Ju et al. examined the in vivo pretreatment with CANA for its potential to prevent post-resuscitation-induced cardiac dysfunction [126]. Mice treated with canagliflozin showed improved neurological score, a shorter time for spontaneous circulation to recover, and a greater survival rate. Increased left ventricular ejection fraction and fractional shortening demonstrated that CANA was beneficial in reversing cardiac arrest and resuscitation-associated cardiac dysfunction. Moreover, CANA improved the systemic inflammatory response and decreased the risk of arrhythmias brought on by early resuscitation. Interestingly, CANA increased cardiac STAT-3 phosphorylation after resuscitation, therefore showing that the cardioprotection is mediated through the STAT-3-dependent cell-survival signaling pathway.

Sabe et al. examined the impact of CANA therapy on myocardial perfusion, contractile function and fibrosis in a large animal model of chronic myocardial ischemia [127]. Compared with the control group, those receiving CANA exhibited increased stroke volume and stroke work alongside a reduced left ventricular stiffness. Notably, CANA was found to be associated with diminished interstitial and perivascular fibrosis in chronically ischemic tissue and to be accompanied by reduced Jak/STAT signaling relative to controls. Within the ischemic myocardium of the CANA-treated group, there was enhanced expression and activation of AMPK, decreased eNOS activation, and unchanged total endothelial NO synthase expression. CANA therapy also mitigated total protein oxidation while boosting the expression of mitochondrial antioxidant superoxide dismutase 2 compared with controls.

### 5.3. Reduction of Cardiac Fibrosis/Adverse Remodeling

Pathophysiology of left ventricular (LV) remodeling comprises both a mechanical component (persistent increase of afterload and/or preload) and a non-mechanical one (neurohumoral stimulation, activation of pro-inflammatory pathway and metabolic remodeling due to mitochondrial dysfunction and suboptimal energy generation). Adverse LV remodeling involves complex interactions between cellular and extracellular components, with collagen accumulation and cross-linking in the extracellular matrix, and activation of signaling pathways promoting interstitial and replacement fibrosis (mainly post-myocardial infarction), all leading to HF progression [128]. The structural changes are responsible for cardiac stiffness and diastolic dysfunction in the setting of HFpEF (typical in diabetes), followed in evolution by the increased wall stress, cavity enlargement and systolic dysfunction as characteristics of HFrEF; therefore, suppressing fibrosis and preventing/reversal of remodeling is an important therapeutic target and SGLT2i are feasible candidates.

#### 5.3.1. Empagliflozin

Li et al. reported, in a non-diabetic model of HF due to pressure overload (TAC), that EMPA attenuated adverse cardiac remodeling independent of reductions in glycemia or diuresis. Moreover, EMPA had direct effects in improving cardiomyocyte contractility and calcium transients in isolated cardiomyocytes. The drug directly binds cardiac glucose transporters, reduced glycolysis, and rebalanced the coupling between glycolysis and oxidative phosphorylation; in terms of signal transduction, it restored the activation of AMPK and inhibited the activation of the mammalian target of rapamycin complex 1 (mTORC1) in failing hearts [110].

Song et al. investigated whether EMPA (2 weeks) administration has a role in ameliorating adverse remodeling in a mouse model of permanent coronary artery ligation; they reported less fibrosis, improved EF and fractional shorting in the treated group. In differentiated H9C2 cardiomyocytes, acute incubation (24 h) with EMPA resulted in an increase in basal and maximal respiratory capacity and also of mitochondrial biogenesis [129].

In light of these considerations, Daud et al. studied the effect of EMPA on myocardial fibrosis and ventricular remodeling early after myocardial infarction in non-diabetic rats. Early post-MI EMPA treatment reduced cardiac fibrosis and attenuated collagen deposition by inhibiting the expression of the fibrotic TGF-1/Smad3 pathway [130]. Additionally, data from another study confirms the benefits of EMPA in reducing the predisposition to lethal ventricular arrhythmia and sudden cardiac death induced by reperfusion injury after acute myocardial infarction [131].

Bruckert et al. evaluated the role of the chronic administration of EMPA in alleviating both cardiac and vascular dysfunction in male rats with Ang II-induced hypertension. In the non-treated hearts, Ang II treatment increased LV mass, fibrosis, collagen I and local infiltration of macrophages and induced diastolic dysfunction. EMPA attenuated both cardiac dysfunction and structural remodeling but did not affect the Ang II-induced hypertension [132].

Santos-Gallego et al. investigated the effects of chronic (2 months) inhibition of SGLT2 with EMPA on ventricular remodeling in the non-diabetic porcine model of post-infarction and reported the alleviation of LV adverse remodeling (lower ventricular mass, reduced dilatation, and less sphericity) plus improvement of LV systolic function (increased EF) and myocardial energetics vs. control animals [90]. Juan Badimon et al. further assessed, in the same model, whether 2 months treatment with EMPA can also alleviate diastolic dysfunction. In an elegant study they demonstrated that EMPA improved diastolic function assessed by 3D echocardiography, invasive hemodynamics and cardiac magnetic resonance through a reduction in myocardial interstitial fibrosis and relaxation of isolated cardiomyocytes through a reduction of cardiac stiffness (assessed by titin phosphorylation). Additionally, EMPA improved NO–cGMP–PKG signaling by increasing eNOS activity, NO availability, cGMP content, and PKG signaling [133].

Kang et al. used cultured human cardiac fibroblasts isolated from atrial tissue harvested during open heart surgery and reported a direct effect of EMPA on both their phenotype (which were smaller and had shorter and fewer extensions, thus suggesting a quiescent phenotype) and function (attenuation of activity, profibrotic markers and collagen remodeling), further supporting the role of EMPA in preventing cardiac remodeling progression [134].

#### 5.3.2. Dapagliflozin

Early studies have unequivocally demonstrated that inflammatory response and a dysfunctional NO–cGMP–PKG signaling pathway in the myocardium play an important role in the LV diastolic dysfunction from HFpEF [135].

Shi et al. investigated the role of chronic DAPA treatment in TAC pressure overload-induced cardiac remodeling and reported the improvement in cardiac systolic function, reduction in myocardial hypertrophy, interstitial and perivascular fibrosis and cardiomyocyte apoptosis. They demonstrated that the beneficial effects on remodeling were mediated via the inhibition by the DAPA of P38, JNK and FoxO1 phosphorylation (expression of phosphorylated proteins was markedly decreased) [120].

In a porcine model of HFpEF, Zhang et al. demonstrated that DAPA decreased blood pressure, attenuated LV concentric remodeling (by reducing proinflammatory cytokines, IL-6 and TNF-α) but failed to improve diastolic function and ventricular compliance. Additionally, the reduced eNOS and PKG-1 protein expression and cGMP content in the aortas of HFpEF pigs were significantly restored after 9 weeks of DAPA treatment [136].

Zhang et al. studied the effects of chronic perfusion with Ang II (4 weeks via minipumps) in non-diabetic rats and showed that DAPA ameliorated LV dysfunction without changing serum glucose or blood pressure. Additionally, the AngII-induced myocardial hypertrophy and fibrosis were attenuated by decreasing collagen synthesis via the inhibition of TGF-β1/Smad signaling [137].

In order to assess whether SGLT2i may induce the reversal of left ventricular remodeling in the clinical arena, Dhingra et al. conducted a meta-analysis of the randomized controlled trials evaluating cardiac remodeling by cardiac magnetic resonance imaging, the authors observed that both EMPA and DAPA are associated with a significant reduction in left ventricular mass in patients with HFrEF, regardless of the presence of diabetes [138].

#### 5.3.3. Canagliflozin

Using Dahl salt-sensitive rats exposed to a high-salt diet that generates hypertension and the standard HFpEF, Ma et al. investigated the effect of CANA, showing the alleviation of cardiac remodeling and LV diastolic dysfunction in these animals. They further demonstrated that ferroptosis, cell death resulting from the iron-dependent generation of lipid peroxides and a significant increase in ROS, was blocked following the CANA treatment [139].

With regard to arrhythmias, Nishinarita et al. provided the first experimental documentation of the suppressive effects of CANA on atrial remodeling in a canine model of pacing-induced atrial fibrillation. CANA treatment suppressed atrial fibrillation inducibility, shortened atrial effective refractory period and decreased conduction velocity, effects that are associated with the suppression of tissue fibrosis and oxidative stress, thus preventing atrial remodeling, the substrate of atrial fibrillation [140].

The “off-target” protective effects of the main SGLT2i are further summarized, as follows: EMPA in Table 1, DAPA in Table 2, and CANA in Table 3.

## 6. Cardiac Protective Effects of Sotagliflozin, a Dual SGLT2/1 Inhibitor

Sotagliflozin (SOTA) is a dual sodium–glucose cotransporter 2 and 1 (SGLT2/1) inhibitor used to treat both type 1 and type 2 diabetes. Sotagliflozin inhibits the renal SGLT2 and intestine SGLT1, delaying glucose absorption and hence lowering postprandial glucose [142]. When compared with SGLT2 inhibitors, dual SGLT1/2 inhibition is a relatively novel pharmacological treatment, hence the number of mechanistic studies is rather limited [143]. Interestingly, a higher SGLT1 expression has been recorded in the human heart than in the kidney; as such, the altered expression of cardiac SGLT1 in diseased myocardium has increased interest in SGLT1/2 compounds [144].

### 6.1. Cardiac Function Enhancement and Arrhythmia Prevention

In a rat model of HFpEF linked to the metabolic syndrome, Bode et al. examined the effects of long-term administration of SOTA on left atrial (LA) remodeling and cellular arrhythmogenesis, such as atrial cardiomyopathy. The dual SGLT2/1 inhibitor improved LA remodeling in vivo, while, in vitro, it enhanced specific aspects of Ca^2+^-mediated cellular arrhythmogenesis, such as the magnitude of spontaneous SR Ca^2+^ release events, the capacity of the mitochondrial Ca^2+^ buffer, diastolic Ca^2+^ buildup, and sodium–calcium exchanger (NCX) activity [145].

### 6.2. Reduction of Cardiac Fibrosis/Adverse Remodeling

SOTA therapy reduced mechanical pressure overload-induced ventricular hypertrophy and the histological indicators of cardiac fibrosis in normoglycemic mice [146]. This was the first study to show a cardioprotective benefit with simultaneous SGLT1/2 inhibition at therapeutic levels, while it was linked to significant diuresis and glucosuria. Interestingly, animals on a modestly hyperglycemic high-fat diet with proximal tubular damage did not experience these advantages [146].

Recent research conducted by Zhong et al. demonstrated the potential cardioprotective benefits of SOTA against myocardial infarction. SOTA enhanced cardiac function and decreased the extent of the infarction in the post-MI rats. Furthermore, SOTA mitigates cardiac remodeling, as evidenced by reduced cardiac hypertrophy and apoptosis [147]. FDA approved SOTA for the treatment of heart failure in diabetic patients in May 2023. The beneficial effects of SOTA in non-diabetic animals are presented below (Table 4).

## 7. SGLT1 Inhibition and Cardioprotection

In contrast with SGLT2, it has been reported, more than one decade ago, that SGLT1 is expressed in the human heart (10-fold higher than that observed in kidney tissue) [148] and is significantly upregulated in the setting of hypertrophic [46,47] and ischemic cardiomyopathy [48]. In the ischemic and hypertrophied human hearts the upregulation of SGLT1 is associated with increased phosphorylation of AMPK, but also with the extracellular-signal regulated kinase 1 and 2 (ERK-1/2) and mammalian target of rapamycin (mTOR) [46].

More recently, Radovits and Merkely et al. reported that both gene and protein SGLT1 expressions were significantly (and comparably) upregulated at the level of cardiomyocytes in patients with HF due to idiopathic dilated cardiomyopathy, ischemic heart disease without and with diabetes, though, interestingly, not in hypertrophic cardiomyopathy. They also showed that the SGLT1 mRNA and protein expressions in the left ventricle positively correlated with the LV end-diastolic diameter and AMPKα phosphorylation, and negatively correlated with the EF and ERK1/2 phosphorylation [149]. Notably, the translational value of their study is supported by the observation that the positive association between the SGLT1 protein expression and AMPKα phosphorylation was initially reported in the mice model of TAC-induced hypertrophy [150].

In the recent years, an increasing number of experimental studies investigated whether deletion or inhibition of SGLT1 provide cardioprotection and addressed the related signal transduction (for a recent review see ref. [151]).

Banerjee et al. reported the presence of SGLT1 in murine cardiomyocyte sarcolemma and the fact that its mRNA cardiac expression increased in WT mice subjected to in vivo coronary artery ligation. Interestingly, in these animals leptin (but not insulin) increased cardiac SGLT1 expression approximately seven-fold relative to control [48], an observation that needs to be recapitulated in humans.

Li et al. have reported that the hearts of mice with cardiomyocyte-specific SGLT1 knockdown are protected from ischemia/reperfusion injury both in vivo and ex vivo with a notable decrease in infarct size, necrosis, and oxidative stress. In cells, AMPK activation increased SGLT1 expression; in turn, SGLT1 overexpression activated PKC and NADPH oxidase 2 (Nox2) [152].

Chai et al. assessed the role of SGLT1 in rat H9c2 cardiomyocytes subjected to various glucose concentrations and the associated mechanistic pathways. They report that SGLT1 knockdown restores cell proliferation, mitigates cytotoxicity elicited by exposure to fluctuating glucose, and reduces oxidative stress by decreasing ROS and enhancing antioxidant activity. Additionally, SGLT1 knockdown attenuates mitochondrial dysfunction by preserving mitochondrial membrane potential, promoting mitochondrial fusion, and reducing apoptotic cell death, collectively suggesting its role in alleviating the cardiomyocyte dysfunction induced by fluctuating glucose levels [153].

Human mutations in the gene PRKAG2 encoding the gamma2 subunit of AMPK have been reported to cause a glycogen storage cardiomyopathy. Banerjee et al. investigated the cardiac SGLT1 expression in transgenic mice with the human T400N mutation (TG T400N), which is characterized by inappropriate AMPK activation and subsequent glycogen storage in the heart. These authors report that increased AMPK activity is responsible for the increased mRNA and protein expression of SGLT1 that further increased glucose uptake in PRKAG2 cardiomyopathy [154]. In a comprehensive study in transgenic mice, Ramratnam et al. further dissected the role of SGLT1 in the pathogenesis of PRKAG2 cardiomyopathy. They report that cardiac SGLT1 knockdown in this model led to a reduction in glycogen storage, mitigated myocardial hypertrophy, and alleviated left ventricular dysfunction [155].

Elevated levels of SGLT1 gene expression have been linked to cardiac hypertrophy. Thus, Matsushita et al. investigated whether pressure overload could enhance SGLT1 gene expression, thus contributing to the development of hypertrophic cardiomyopathy. Their study revealed that chronic pressure overload results in decreased left ventricular fractional shortening and left ventricular dilatation in transverse aortic constriction (TAC)-operated wildtype (WT) mice, but not in TAC-operated SGLT1−/− mice. In TAC-operated WT mice, increased gene expression of SGLT1, atrial natriuretic peptide, brain natriuretic peptide, IL-18, connective tissue growth factor, and collagen I was observed. Conversely, TAC-operated SGLT1−/− mice did not exhibit elevated expression of these factors [156].

Sawa et al. investigated the effect of KGA-2727, a selective SGLT1 inhibitor, in mice with myocardial infarction (MI)-induced ischemic cardiomyopathy. MI was induced by left anterior descending coronary artery (LAD) ligation, with or without KGA-2727 administration in C57BL/6J mice. After four weeks, LV fractional shortening was significantly improved by KGA-2727 in mice with MI. Cardiomyocyte diameter and the LV gene expressions of ANP, BNP, β-MHC, and IL-18 were elevated in LAD-ligated as compared with sham-operated mice, and KGA-2727 inhibited these increases. Additionally, LV fibrosis was increased in LAD-ligated mice as compared with sham-operated mice, and KGA-2727 decreased it in LAD-ligated left ventricles. Regardless of KGA-2727 treatment, SGLT1 protein expression was significantly higher in LAD-ligated mouse ventricles. These findings suggest that KGA-2727 pretreatment protected against MI-induced left ventricular remodeling by blocking SGLT1, potentially offering a new therapeutic approach for ischemia-induced cardiomyopathy [157]. Notably, in mice, cardiac SGLT1 expression appears to increase with age [151]. The question of whether SGLT1 expression increases with age in humans is worth further investigation.

More recently, Sayour et al. have published an elegant study on the relation between the ventricular SGLT1 expression and the magnitude of nitro-oxidative stress in two non-diabetic rat models of chronic HF elicited by pressure and volume overload. They showed that upregulation of the SGLT1 protein occurs irrespective of the type of chronic hemodynamic overload and is significantly correlated with the increased ventricular expression of Nox4, 4-hydroxy-2-nonenal and 3-nitrotyrosine. Interestingly, the AMPKα activity was significantly reduced in both models [158].

Finally, Dasari et al. addressed the in vitro roles of CANA and DAPA in mitigating the deleterious effects of glucolipotoxicity elicited by exposing H9C2 cardiomyocytes to high glucose (HG) and palmitic acid (PA). They report that no expression of SGLT2 was detected in control as well as in HG and PA and SGLT2i treated cardiomyocytes. In contrast, a time-dependent significant increase at 24, 48, and 72 h was reported for SGLT1 expression after exposure to HG and PA. The authors conclude that both CANA and DAPA decreased the glucolipotoxicity-related oxidative stress and apoptosis via SGLT1 inhibition in cardiomyocytes [159].

The cardioprotective effects of SGLT1 inhibition described in experimental settings are summarized in Table 5.

## 8. Conclusions and Future Perspectives

Despite significant advances in the standards of care for HF, particularly in the case of HFpEF and HFmrEF, the current therapeutic approaches have not always been successful in averting clinical deterioration. The SGLT2i are currently recognized as the “statins of the 21st century” [160] and their class effect on the cardiovascular outcomes has been unequivocally confirmed for the primary endpoint, the composite of hospitalization for HF or cardiovascular mortality [161]. Their use in clinical practice has improved both cardiovascular and renal outcomes in an unanticipated way, despite the fact that the relevance of composite endpoints has been the topic of a critical review [162].

There is an unmet need for the elucidation of the mechanisms underlying their relative rapid efficacy in providing cardioprotection and to assess whether the expression of SGLT2 receptor will be identified/increase with the age of the diseased human heart.

In this respect, a concerted translational approach, using both cardiac and vascular human samples, will be able to confirm that a peculiar mechanism is consistent in the cardiovascular system and among the gliflozins approved for HF therapy at concentrations relevant for the clinical arena. Whether this mechanism can also be relevant for kidney protection is equally important, as the pathophysiological relationship between the kidneys and the heart is bidirectional, with CKD and HF frequently causing or exacerbating the condition of the other [21].

Lastly, their interference with the occurrence/magnitude of SGLT1 inhibition needs to be further explored as a potential contributor to cardiac protection. This is particularly important in the setting of cardiac pathologies, with SGLT1 expression being reported to have increased in all cardio-metabolic pathologies.

Nevertheless, gliflozins will remain at the forefront of scientific research because of their partially elucidated off-target effects, which will continue to broaden the therapeutic scenario. In line with this, the most recent research is exploring the potential application of SGLT2 inhibitors in cardio-oncology, i.e., to alleviate the cardiovascular side effects of chemotherapy.

## Figures and Tables

**Table 1:** Summary of the cardioprotective effects of EMPA described in cell lines, non-diabetic animal models and human samples.

Model	Drug Dose	Effects	Reference
**1. CELLULAR AND MOLECULAR EFFECTS**			
**1.1. Protection of Mitochondrial Function and Structure**			
H9C2 cardiomyocytes	1 µM for 24 h	▪ Improved basal and maximal respiratory capacity ▪ Increased various markers for mitochondrial content	[129]
Murine isolated cardiac microvascular endothelial cells	10 mg/kg/day 7 days prior to an I/R myocardial injury	▪ Normalized mitochondrial fission and fusion ▪ Activation of mitophagy ▪ Neutralized ROS production suppressed mitochondrial apoptosis	[61]
C57Bl/6J mice	10 mg/kg/day for 4 weeks	In vivo: ▪ Improved OXPHOS ▪ Enhanced mitochondrial biogenesis ▪ Restored normal mitochondria morphology ▪ Enhanced autophagy ▪ Decreased cardiac apoptosis ▪ Inhibited cardiac ROS production ▪ Upregulated expression of endogenous antioxidants Ex vivo: ▪ Increased mitochondrial respiration of cardiac fibers	[59]
C57BL/6J mice	For 8 weeks, starting at 4 weeks post operation (ligation of the left anterior descending coronary artery)	▪ Inhibited mitochondrial fission ▪ mdivi1-mediated attenuation of mitochondria dysfunction ▪ Increased ATP production	[62]
C57BL/6J mice	10 mg/kg/day for 2 weeks	▪ Increased baseline mitochondrial oxygen consumption and maximal respiratory capacity ▪ Increased mitochondrial proteins, including representative subunits of each of the 5 OXPHOS complexes, as well as of TOM20 and Mfn2	[129]
Male mice deficient for heart and skeletal muscle-specific MnSOD	10 mg/kg for 7 weeks	▪ Upregulated OXPHOS capacity ▪ Decreased glycolysis (blood lactate)	[60]
Sprague–Dawley rats	30 mg/kg/day, starting before surgery (EMPA—early) or 2 weeks after surgery (EMPA—late)	▪ Stimulated mitochondrial biogenesis ▪ Increased cardiac ATP production	[88]
Yorkshire pigs	10 mg for 2 months	▪ Increased myocardial ATP content	[90]
**1.2. Alleviation of Oxidative Stress and Inflammation**			
LPS-treated HL-1 (mouse)cardiomyocytes	0.2–2 µM	▪ Attenuated LPS-induced TNFα and iNOS expression ▪ Activated AMPK ▪ ATP depletion prevention	[74]
HL-1 (mouse) cardiomyocytes	50, 100 and 500 nM for 24 h	▪ Increased cell viability ▪ Decreased ROS ▪ Decreased lipid peroxidation	[75]
Primary cardiomyocytes from neonatal rats	10 µM	▪ Decreased oxidative stress and apoptosis ▪ Increased p-AMPK and PGC-1α protein ▪ Decreased p-MYPT1	[76]
C57Bl/6 mice (HFrEF model); Dahl salt-sensitive rats (HFpEF model)	10 mg/kg per day for 2 weeks	▪ Attenuated activation of the NLRP3 inflammasome ▪ Attenuated expression of associated markers of sterile inflammation	[73]
C57Bl6/N mice model of endotoxaemia	5 mg/kg i.p. for 8 h	▪ Lower plasma levels of TNFα	[74]
C57BL/6J mice treated with doxorubicin	10 mg/kg/day for 10 days	▪ Reduced ferroptosis and apoptosis ▪ Reduced xanthine oxidase expression ▪ Decreased inflammation engaging NLRP3- and MyD88-related pathways ▪ Increased EF	[75]
Sprague–Dawley rats	30 mg/kg/day starting before surgery (EMPA—early) or 2 weeks after surgery (EMPA—late)	▪ Reduced myocardial oxidative stress	[88]
Skinned fibers prepared from LV biopsies, harvested from patients with HFpEF	5 µM for 60 min	▪ Decreased the levels of H_2_O_2_, 3-nitrotyrosine, glutathione (GSH) and lipid peroxidation in both cytosol and mitochondria ▪ Reduced PKGIα oxidation and increased its activity ▪ Reduced markers of microvascular inflammation: ICAM-1, VCAM-1, TNF-a, and IL-6	[77]
**2. WHOLE-ORGAN EFFECTS**			
**2.1. Improvement of Cardiac Metabolism**			
Langendorff rat heart	10 µM after 10 min of baseline perfusion and maintained in the perfusion buffer throughout the entire protocol of I/R	▪ Increased ATP and PCr levels ▪ Decreased 13C1 glucose incorporation ▪ Increased 13C4 βHb incorporation into the TCA cycle intermediates succinate, citrate, and glutamate	[89]
C57Bl6/N mice model of endotoxaemia	5 mg/kg i.p. for 8 h	▪ Cardiac AMPK phosphorylation	[74]
Sprague–Dawley rats	30 mg/kg/day starting before surgery (EMPA—early) or 2 weeks after surgery (EMPA—late)	▪ Normalized myocardial uptake and oxidation of glucose and fatty acids ▪ Increased circulating ketone levels and myocardial expression of the ketone body transporter and two critical ketogenic enzymes: succinyl-CoA:3-ketoacid CoA transferase and *β*-hydroxybutyrate dehydrogenase	[88]
Yorkshire pigs	10 mg for 2 months	▪ Shift in myocardial metabolism from glucose oxidation to that of ketone bodies and free fatty acids	[90]
**2.2. Cardiac Function Enhancement and Arrhythmia Prevention**			
C57Bl/6 mice	10 mg/kg/day for 2 weeks	▪ Improved systolic (but not diastolic) function	[106]
C57Bl6/N mice model of endotoxaemia	5 mg/kg i.p. for 8h	▪ Preserved cardiac function ▪ Reduced cardiac iNOS ▪ Lower plasma levels of creatine kinase MB	[74]
C57BL/6J mice	10 mg/kg/day for 4 weeks, starting 2 weeks after TAC	▪ Alleviated LV systolic and diastolic dysfunction ▪ Increased exercise endurance	[110]
Langendorff rat heart	10 µM after 10 min of baseline perfusion and maintained in the perfusion buffer throughout the entire protocol of I/R	▪ Improved left ventricular-developed pressure	[89]
Sprague–Dawley rats	30 mg/kg/day, starting before surgery (EMPA—early) or 2 weeks after surgery (EMPA—late)	▪ Increased LV ejection fraction	[88]
Sprague–Dawley rats	0.35 mg/g mixed in chow for 4 weeks	▪ Improved both wall stress and diastolic function	[108]
Sprague–Dawley rats	20 mg/kg/day for 7 days before I/R	▪ Decreased myocardial vulnerability to sudden cardiac death ▪ Decreased susceptibility to reperfusion-induced arrhythmias ▪ Increased cardiac ERK1/2 phosphorylation postreperfusion	[131]
SHR rats	20 mg/kg/day for 12 weeks	▪ Decreased BP ▪ Alleviated systolic dysfunction ▪ Restored atrial and ventricular expression of PPARα, ACADM, natriuretic peptides, and TNF-α	[107]
Fischer F344 rats	20 mg/kg/day for 6 weeks	▪ Increased load-independent measures of cardiac contractility, preload recruitable stroke work and end-systolic pressure volume relationship ▪ Enhanced cardiac performance in the heart failure setting ▪ Increased systolic blood pressure ▪ Reduced LV end-diastolic pressure	[109]
Male normotensive normoglycemic Wistar rats	1 mg/kg/day for 1 week	▪ Increased ventricular systolic pressure, mean pressure, and the max dP/dt ▪ Increased cardiac output, stroke volume, and fractional shortening ▪ Reduced MMP9 in peri-infarct tissue ▪ Favorably regulated cardiac transporters SERCA2a and NHE1 in the peri-infarct tissue	[111]
Male Wistar rats	300 mg empagliflozin/kg chow for 5 weeks	▪ Decreased right ventricular end-systolic pressures	[113]
Wistar rats	30 mg/kg/day for 5 weeks	▪ Attenuated the AngII-induced diastolic dysfunction	[132]
Yorkshire pigs	10 mg for 2 months	▪ Improved myocardial work efficiency ▪ Alleviated remodeling and LV systolic function post MI	[90]
Female Yorkshire non-diabetic pigs	10 mg/day for 2 months	▪ Improved diastolic function ▪ Reduced interstitial myocardial fibrosis ▪ Improved nitric oxide signaling (eNOS activity, NO availability, cGMP content, PKG signaling) ▪ Enhanced titin phosphorylation	[133]
Ventricular biopsies from patients with HFpEF and ventricular trabeculae isolated from explanted hearts of patients with end-stage HFrEF	0.1, 0.5 and 1 µmol/L (for 30 min or 60 min)	▪ Reduced myofilament passive stiffness ▪ Enhanced phosphorylation levels of myofilament regulatory proteins: TnI, MyBPC, titin ▪ Reduction of abnormally increased diastolic tension	[112]
**2.3. Reduction of Cardiac Fibrosis/** **Adverse Remodeling**			
C57BL/6J wildtype male mice	10 mg/kg/day for 2 weeks	▪ Reduced infiltration of immune cells and fibrosis in the border zone region ▪ Better EF and FS obtained by echocardiography	[129]
C57BL/6J mice	10 mg/kg/day for 4 weeks starting 2 weeks after TAC	▪ Attenuated adverse ventricular remodeling and cardiac fibrosis	[110]
C57BL/6J mice	For 8 weeks, starting at 4 weeks post operation (ligation of the left anterior descending coronary artery)	▪ Alleviated myocardial fibrosis and cardiac dysfunction	[62]
SHR rats	20 mg/kg/day for 12 weeks	▪ Attenuated cardiac fibrosis	[107]
Sprague–Dawley rats	30 mg/kg/day for 4 weeks	▪ Reduced myocardial fibrosis ▪ Reduced collagen deposition after MI	[130]
Sprague–Dawley rats	30 mg/kg/day, starting before surgery (EMPA—early) or 2 weeks after surgery (EMPA—late)	▪ Attenuated cardiomyocyte hypertrophy ▪ Diminished interstitial fibrosis	[88]
Sprague–Dawley rats	0.35 mg/g mixed in chow for 4 weeks	▪ Reduced left ventricular mass	[108]
Wistar rats	300 mg empagliflozin/kg chow for 5 weeks	▪ Reduced hypertrophy	[113]
Wistar rats	30 mg/kg/day for 5 weeks	▪ Prevented pro-fibrotic and pro-remodeling responses, cardiomyocyte hypertrophy and macrophage infiltration in the left ventricle of Ang II-treated rats	[132]
Cardiac fibroblasts cultured from human atrial tissue harvested during open heart surgery	0–5 µM for 72 h	▪ Attenuated TGFβ1-induced fibroblast activation ▪ Smaller myofibroblasts in size with shorter and fewer number of extensions ▪ Attenuated ECM remodeling as measured by collagen fiber alignment index ▪ Suppressed expression of various profibrotic markers	[134]

Abbreviations: ACADM, acyl-CoA dehydrogenase medium chain; AMPK, adenosine monophosphate-activated protein kinase; BP, blood pressure; cGMP, cyclic guanosine monophosphate; EF, ejection fraction; eNOS, endothelial nitric oxide synthase; ECM, extracellular matrix; ERK1/2, extracellular signal-regulated kinases; FS, fractional shortening; I/R, ischemia/reperfusion; LV, left ventricle; LPS, lipopolysaccharide; Mfn2, mitofusion 2; MI, myocardial infarction; MMP, matrix metalloproteinase; MYPT1, myosin phosphatase target subunit 1; NHE1, sodium–hydrogen exchanger 1; NO, nitric oxide; OXPHOS, five complexes of oxidative phosphorylation; PGC-1α, peroxisome proliferator receptor gamma coactivator 1 alpha; PKG, protein kinase G; PPARα, peroxisome proliferator-activated receptor alpha; ROS, reactive oxygen species; SERCA2a, sarcoplasmic/endoplasmic reticulum calcium ATPase; TNF-α, tumor necrosis factor alpha; TOM, preprotein translocase of the mitochondrial outer membrane; MyBPC, myosin binding protein C; NLRP3, NLR family pyrin domain containing 3; TNFα, tumor necrosis factor alpha; TnI, troponin I; IL, interleukin; ICAM-1, intercellular adhesion molecule 1; VCAM-1, vascular cell adhesion molecule 1.

**Table 2:** Summary of the cardioprotective effects of Dapagliflozin described in cell lines, non-diabetic animal models and human samples.

Model	Drug Dose	Effects	Reference
**1. CELLULAR AND MOLECULAR EFFECTS**			
**1.1. Protection of Mitochondrial Function and Structure**			
Cardiac H9c2 myoblasts treated with doxorubicin	0–20 µM	▪ Improved mitochondrial dysfunction via Nrf2	[79]
H9c2 cardiac myoblasts	0–4 µM	▪ Prevented the H/R-induced abnormal PGC-1α expression, mitochondrial membrane potential, and mitochondrial DNA copy number	[78]
Primary neonatal cardiomyocytes (Sprague–Dawley rats)	1 h hypoxia/4 h reoxygenation (H/R)		
C57BL/6 mice	1 mg/kg/day for 14 weeks	▪ Improved mitochondrial dynamics and function	[63]
Wistar rats	1 mg/kg given pre-ischemia, at the time of ischemia and at the beginning of reperfusion	▪ Improved mitochondrial function	[123]
**1.2. Alleviation of Oxidative Stress and Inflammation**			
Cardiac H9c2 myoblasts treated with doxorubicin	0–20 µM	▪ Induced activation of AKT/PI3K signaling ▪ Upregulated antioxidants HO-1, NQO1 and SOD ▪ Reduced oxidative stress ▪ Inhibition of IL-8 increase	[79]
H9c2 cardiac myoblasts	0–4 µM	▪ Reduced PKC phosphorylation ▪ Diminished the H/R-elicited oxidative stress via the AMPK/PKC/Nox2 pathway	[78]
Murine ventricular cardiomyocytes	1 μM for 10 min	▪ Reduced cardiac inflammation	[121]
C57BL/6 mice	1 mg/kg/day for 14 weeks	▪ Suppressed ROS production	[63]
Sprague–Dawley rats	10 mg/kg/day for 2 weeks	▪ Prevented the development inflammatory infiltrates	[81]
Landrace pigs	10 mg/day for 9 weeks	▪ Inhibited IL-6 and TNF-α in aortic tissues	[136]
**2. WHOLE ORGAN EFFECTS**			
**2.1. Improvement of Cardiac Metabolism**			
H9c2 cardiac myoblasts	0–4 µM	▪ Increased phosphorylated AMPK	[78]
C57BL6/N mice Isolated working hearts	4 weeks administration after TAC acute exposure to 0.2 mM or 0.6 mM βOHB	▪ Increased βOHB oxidation rates in TAC DAPA hearts ▪ Increased ATP production at both βOHB concentrations by increasing the contribution of glucose oxidation to ATP production	[102]
**2.2. Cardiac Function Enhancement and Arrhythmia Prevention**			
H9c2 cardiac myoblasts	0–4 µM	▪ Reversed the H/R-induced apoptosis ▪ Decreased apoptosis	[78]
Primary neonatal cardiomyocytes (Sprague–Dawley rats)	1 h hypoxia/4 h reoxygenation (H/R)		
Murine ventricular cardiomyocytes	1 μM for 10 min	▪ Reduced diastolic Ca^2+^ and Na^+^ overload ▪ Increased Ca^2+^ transient amplitude ▪ Reduced endothelial activation and endothelial NOS deficiency	[121]
Porcine atrial cardiomyocytes	100 µmol/L for up to 15 min	▪ Atrial-dominant reduction of myocardial conduction velocity	[124]
C57BL/6 mice	1 mg/kg/day for 14 weeks	▪ Improved cardiac function and structure	[63]
C57BL/6J mice	1 mg/kg/day for 4 weeks	▪ Improved cardiac systolic function ▪ Reduced myocyte apoptosis	[120]
SGLT2 full-KO mice	1.50 mg/kg/day 3 days post-MI surgery and 2 weeks after the TAC surgery	▪ Reduced left ventricular end-systolic diameter ▪ Enhanced left ventricular EF ▪ Enhanced left ventricular FS ▪ Increased cardiac output ▪ Reduced left ventricular end-diastolic/systolic volume ▪ Increased maximum rate of left ventricular pressure rise/decay ▪ Reduced heart weight to body weight ratio and heart weight to tibial length ratio ▪ Reduced transcription of heart failure markers (ANP and BNP) in left ventricular heart tissue ▪ Reduced left ventricular collagen fraction ▪ Reduced transcription of fibrosis in left ventricular heart tissue ▪ Increased remaining number of cardiomyocytes post MI ▪ Reduced compensatory myocardial contraction	[80]
Schlager mice (BPH/2J strain)	40 mg/kg every 2 days for 2 weeks	▪ Reduced elevations of tyrosine hydroxylase and norepinephrine levels in kidneys	[82]
Dahl rats	0.1 mg/kg/day for 6 weeks	▪ Improved diastolic function ▪ BP remained elevated	[121]
Sprague–Dawley rats	10 mg/kg/day for 2 weeks	▪ Prevented tyrosine hydroxylase overexpression in Ang II-treated rats	[81]
Sprague–Dawley rats	10 mg/kg/day for 6 weeks	▪ Reduced left ventricular dysfunction ▪ Restored the end-systolic pressure-volume relationship ▪ Decreased the inducibility and duration of atrial fibrillation	[122]
Wistar rats	1 mg/kg given pre-ischemia, at the time of ischemia and at the beginning of reperfusion	▪ Increased LV function ▪ Attenuated infarct size ▪ Reduced cardiac apoptosis	[123]
Landrace pigs	10 mg/day for 9 weeks	▪ Mitigated tyrosine hydroxylase expression and norepinephrine concentration in the aorta ▪ Restored expression of eNOS and the PKG-1 protein and the cGMP content in aortas	[136]
Human atrial cardiomyocytes	10 and 100 µmol/L for up to 10 min	▪ Decreased peak sodium current ▪ Inhibition of the transient outward potassium current	[124]
**2.3. Reduction of Cardiac Fibrosis/Adverse Remodeling**			
Cardiac H9c2 myoblasts treated with doxorubicin	0–20 µM	▪ Downregulated markers of hypertrophy (natriuretic peptides) and fibrosis (phospho-Smad3, collagen I, fibronectin, and α-SMA)	[79]
Murine ventricular cardiomyocytes	1 μM for 10 min	▪ Attenuation of pro-fibrotic signaling	[121]
C57BL/6 mice	1 mg/kg/day for 14 weeks	▪ Reduced myocardial injury and adverse fibrotic remodeling ▪ Reduced apoptosis	[63]
C57BL/6J mice	1 mg/kg/day for 4 weeks	▪ Reduced cardiac hypertrophy, interstitial and perivascular myocardial fibrosis	[120]
SGLT2 full-KO mice	1.50 mg/kg/day 3 days post-MI surgery and 2 weeks after the TAC surgery	▪ Suppressed adverse cardiac hypertrophy	[80]
Sprague–Dawley rats	10 mg/kg/day for 2 weeks	▪ Prevented the development of myocardial hypertrophy and fibrosis	[81]
Sprague–Dawley rats	5 mg/kg/day for 4 weeks	▪ Improved LV dysfunction and cardiac remodeling ▪ Inhibited myocardial hypertrophy fibrosis ▪ Increased collagen synthesis	[137]
Landrace pigs	10 mg/day for 9 weeks	▪ Attenuated heart concentric remodeling	[136]
Cardiac fibroblasts	0.5, 1, 10 μM for 24 h	▪ Suppressed collagen production	[137]

Abbreviations: βOHB, β-hydroxybutyrate; ANP, atrial natriuretic peptide; BP, blood pressure; BNP, brain natriuretic peptide; cGMP, cyclic guanosine monophosphate; eNOS, endothelial nitric oxide synthase; H/R, hypoxia/reoxygenation; HO-1, hemoxygenase-1; IL, interleukin; LV, left ventricle; MI, myocardial infarction; NO, nitric oxide; NOX, NADPH oxidase; NQO1, NAD(P)H dehydrogenase quinone 1; PKG, protein kinase G; ROS, reactive oxygen species; SOD, superoxide dismutase.

**Table 3:** Summary of the cardioprotective effects of Canagliflozin described in cells lines and non-diabetic animal models.

Model	Drug Dose	Effects	Reference
**1. CELLULAR AND MOLECULAR EFFECTS**			
**1.1. Protection of Mitochondrial Function and Structure**			
Yorkshire swine	300 mg/day for 5 weeks	▪ Increased pathways related to glucose metabolism, fatty acid metabolism and oxidative phosphorylation ▪ Increased mitochondrial complexes (I, II, III, IV)	[64]
Yorkshire swine	300 mg/day for 5 weeks	▪ Increased expression of mitochondrial antioxidant SOD 2	[127]
**1.2. Alleviation of Inflammation and Oxidative Stress**			
HL-1 cell line	5 µg/mL for 24 h	▪ Inhibited expression of inflammatory factors COX-2 and iNOS ▪ Regulated inflammation and ferroptosis through AMPK	[141]
Long–Evans rats	5 mg/kg/day for one week	▪ Alleviated oxidative stress ▪ Suppressed the upregulation of Nox4 and iNOS	[83]
Beagle dogs	3 mg/kg/day for 3 weeks	▪ Suppressed oxidative stress	[140]
Yorkshire swine	300 mg/day for 5 weeks	▪ Increased antioxidants (catalase, glutathione-s-transferase, peroxiredoxin-6, and heat shock protein 60)	[64]
Yorkshire swine	300 mg/day for 5 weeks	▪ Decreased expression of fatty acid oxidation inhibitor acetyl-CoA carboxylase and decreased phosphorylated/inactivated acetyl-CoA carboxylase ▪ Modulation in insulin resistance markers *p*-IRS1, *p*-PKCα, and PI3-kinase ▪ Significant increase in inflammatory markers interleukin 6, interleukin 17, interferon-gamma, and inducible nitric oxide synthase	[84]
Yorkshire swine	300 mg/day for 5 weeks	▪ Reduced total protein oxidation	[127]
**2. WHOLE-ORGAN EFFECTS**			
**2.1. Cardiac Function Enhancement and Arrhythmia Prevention**			
C57/BL6 mice	10 mg/kg/day	▪ Increased left ventricular ejection fraction and fractional shortening ▪ Promoted phosphorylation of cardiac STAT-3 post-resuscitation	[126]
Long–Evans rats	5 mg/kg/day for one week	▪ Reduced cardiomyocyte apoptosis ▪ Reduced plasma levels of CK–MB	[83]
Dahl rats (salt sensitive)	20 mg/kg/day for 12 weeks	▪ Improved blood pressure ▪ Improved LV diastolic dysfunction ▪ Inhibited ferroptosis in ventricular tissue	[139]
Beagle dogs	3 mg/kg/day for 3 weeks	▪ Decreased atrial effective refractory period, conduction velocity and AF inductibility	[140]
Lean swine	300 mg 24 h prior to and the morning of an invasive physiologic study protocol	▪ Preserved cardiac contractile function and efficiency during ischemia ▪ Increased EDV volume and cardiac output ▪ Reduced MI size after 60 min of ischemia	[125]
Yorkshire swine	300 mg/day for 5 weeks	▪ Increased cardiac index	[64]
Yorkshire swine	300 mg/day for 5 weeks	▪ Increased stroke volume and stroke work ▪ Reduced JAK/STAT signaling ▪ Increased expression and activation of AMPK ▪ Decreased activation of endothelial nitric oxide synthase	[127]
**2.2. Reduction of Cardiac Fibrosis/Adverse Remodeling**			
Dahl rats (salt sensitive)	20 mg/kg/day for 12 weeks	▪ Improved cardiac remodeling	[139]
Beagle dogs	3 mg/kg/day for 3 weeks	▪ Suppressed atrial remodeling ▪ Suppressed the degree of interstitial fibrosis	[140]
Yorkshire swine	300 mg/day for 5 weeks	▪ Decreased left ventricular stiffness ▪ Decreased interstitial and perivascular fibrosis in chronically ischemic tissue	[127]

Abbreviations: AF, atrial fibrillation; AMPK, adenosine monophosphate-activated protein kinase; CK–MB, creatine kinase–myocardial band; COX-2, cyclooxygenase 2; EDV, end-diastolic volume; iNOS, inducible nitric oxide synthase; LV, left ventricle; NOX, NADPH oxidase; NOX4, NADPH oxidase 4; STAT-3, signal transducer and activator of transcription 3; PKC, protein kinase C.

**Table 4:** Summary of the cardioprotective effects of Sotagliflozin described in non-diabetic animal models.

Model	Drug Dose	Effects	Reference
C57BL/6J mice (on ND)	10 mg/kg for 7 weeks	▪ Attenuated cardiac hypertrophy and histological markers of cardiac fibrosis	[146]
Sprague–Dawley rats	10 mg/kg for 2 weeks	▪ Reduced cardiac hypertrophy ▪ Reduced cardiac apoptosis ▪ Reduced cardiac inflammatory response ▪ Improved cardiac function ▪ Improved cardiac remodeling	[147]

Abbreviations: ND, normal diet.

**Table 5:** Summary of the cardioprotective effects of SGLT1 inhibition described in cell-lines and non-diabetic animal models.

Model	Effects	Reference
**Alleviation of Mitochondrial Dysfunction and Oxidative Stress**		
H9c2 cardiomyocytes subjected to glucose fluctuation	▪ Restored cell proliferation ▪ Suppressed cytotoxicity ▪ Reduced intracellular ROS level ▪ Increased antioxidant activity ▪ Restored mitochondrial membrane potential ▪ Promoted mitochondrial fusion ▪ Downregulated Bax expression ▪ Upregulated Bcl-2 expression ▪ Reduced caspase-3 activation	[153]
Mice with cardiomyocyte-specific knockdown of SGLT1	▪ Decreased oxidative stress	[152]
**Cardiac Function Improvement and Prevention of Remodeling**		
Mice with cardiomyocyte-specific knockdown of SGLT1	▪ Decreased infarct size and necrosis	[152]
Cardiac knockdown of SGLT1 in a murine model of PRKAG2 cardiomyopathy	▪ Decreased markers of cardiac hypertrophy ▪ Decreased cardiac glycogen content ▪ Decreased LV dilation	[155]
SGLT1-deficient (SGLT1−/−) and wildtype (WT) mice	▪ Decreased LV fractional shortening and dilatation in TAC-operated WT mice, but not in TAC-operated SGLT1−/− mice ▪ TAC-operated SGLT1−/− mice did not exhibit elevated expression of SGLT1, ANP, BNP, IL-18, CTGF, and collagen I	[156]
C57BL/6J mice	▪ Improved LV fractional shortening ▪ Inhibited ANP, BNP, β-MHC, and IL-18 genes expression ▪ Decreased myocardial fibrosis ▪ Decreased CTGF and MMP-3 gene expression	[157]

Abbreviations: ANP, atrial natriuretic peptide; β-MHC, myosin heavy chain beta; BNP, brain natriuretic peptide; CTGF, connective tissue growth factor; MMP-3, matrix metalloproteinase 3.

## Data Availability

The article is a narrative review. No data were generated or analyzed.
